# Development of Deletion Lines for Chromosome 3D of Bread Wheat

**DOI:** 10.3389/fpls.2019.01756

**Published:** 2020-01-28

**Authors:** Radim Svačina, Miroslava Karafiátová, Magdaléna Malurová, Heïdi Serra, Dominik Vítek, Takashi R. Endo, Pierre Sourdille, Jan Bartoš

**Affiliations:** ^1^Institute of Experimental Botany, Czech Academy of Sciences, Centre of the Region Hana for Biotechnological and Agricultural Research, Olomouc, Czechia; ^2^INRA, Génétique, Diversité, Ecophysiologie des Céréales, Clermont-Ferrand, France; ^3^Faculty of Agriculture, Ryukoku University, Shiga, Japan

**Keywords:** wheat, deletion line, homoeologous pairing, *Ph2*, gametocidal

## Abstract

The identification of genes of agronomic interest in bread wheat (*Triticum aestivum* L.) is hampered by its allopolyploid nature (2n = 6x = 42; AABBDD) and its very large genome, which is largely covered by transposable elements. However, owing to this complex structure, aneuploid stocks can be developed in which fragments or entire chromosomes are missing, sometimes resulting in visible phenotypes that help in the cloning of affected genes. In this study, the 2C gametocidal chromosome from *Aegilops cylindrica* was used to develop a set of 113 deletion lines for chromosome 3D in the reference cultivar Chinese Spring. Eighty-four markers were used to show that the deletions evenly covered chromosome 3D and ranged from 6.5 to 357 Mb. Cytogenetic analyses confirmed that the physical size of the deletions correlated well with the known molecular size deduced from the reference sequence. This new genetic stock will be useful for positional cloning of genes on chromosome 3D, especially for *Ph2* affecting homoeologous pairing in bread wheat.

## Introduction

Bread wheat (*Triticum aestivum* L.) is one of the most important cultivated crops. It emerged through two distinct hybridization events between three diploid species, resulting in its allohexaploid nature. The genetic material consists of three closely related subgenomes, namely A, B, and D (Huang et al., 2002), which generate the genomic plasticity necessary for bread wheat to grow under a wide range of climatic conditions. Moreover, bread wheat tolerates the creation of aneuploid lines, such as nullisomic, substitution, deletion, and many other types. However, the three sets of homoeologous chromosomes create a vulnerability to incorrect chromosome pairing during meiosis, possibly resulting in aberrant gametes. Therefore, to maintain the pairing behavior during meiosis, a system developed in wheat that is enforced genetically by *pairing homoeologues* (*Ph*) genes. In this control, the most effective genes are *Ph1* and *Ph2. Ph1* is on the 5B chromosome and has a major influence on homoeologous chromosome pairing (Riley and Chapman, 1958; Sears and Okamoto, 1958). *Ph2* is on the short arm of chromosome 3D (Mello-Sampayo, 1971) and has less of an effect compared with *Ph1*. Despite some attempts at positional cloning (Sutton et al., 2003), *Ph2* has not been formally identified to date. Other genes contribute to the control of homoeologous pairing but have only minor influence, such as *Ph3*, which is on the short arm of chromosome 3A and is possibly a homoeologous variant of *Ph2* (Driscoll, 1972; Mello-Sampayo and Canas, 1973).

Genes are usually maintained in a population by benefiting their hosts or alternatively, by high linkage to such a gene (a phenomenon called linkage-drag). However, there are exceptions to this rule, such as transposable elements, B chromosomes, and gametocidal genes/chromosomes. These genetic units use various “selfish” behaviors to either preserve their existence in the population or to increase their number. The gametocidal genes or chromosomes secure their inheritance to progeny through induction of genomic aberrations and consequent total or partial sterility in gametes lacking them. In wheat, this phenomenon is observed in substitution and addition lines with alien chromosomes from the genus *Aegilops*. The backcrossing of hybrids to wheat between the two species does not remove certain chromosomes of *Aegilops* from the genome of progeny (Endo and Tsunewaki, 1975; Maan, 1975), and chromosomal aberrations are observed in some gametes of such hybrids (Finch et al., 1984). Gametocidal chromosomes originate from the *Aegilops* genomes C, S, and M, and the magnitude of their effect in wheat varies with the type of gametocidal chromosome and the genotype of the wheat background. Whereas some chromosomes cause complete sterility of gametes that lack them (e.g., 2S^lo^, 2S^sh^, T2B-2S^sp.au^, 4S^lo^, 4S^sh^, and 4S^sh^#2); others generate only semi-lethal changes and make it possible to transfer the aberrations to progeny (Endo, 1990; Endo, 2007).

The 2C gametocidal chromosome from *Aegilops cylindrica* has been introduced to the *T. aestivum* ‘Chinese Spring’ background and is being exploited to create mostly terminal deletions of wheat chromosomes. Hereafter, this procedure will be called the “2C gametocidal system” (Endo and Gill, 1996). Tsujimoto (1993) showed that telomeric regions are quickly rebuilt after chromosome breakage and that chromosome stability allows this system to be used as a genetic tool. Endo and Gill (1996) produced 436 deletion lines across all chromosomes using this approach, with subsequent establishment of deletion chromosomes in homozygous/hemizygous constitutions. This resource has been a powerful tool in mapping the position of various genes and markers (Sourdille et al., 2004).

The 2C gametocidal system can be used to create a series of aberrations in any chromosome of wheat. However, the judicious use of existing aneuploid stocks can increase the efficiency and ease in selecting aberrations targeting specific chromosomes. If the monosomic addition line 2C is crossed as male to a nulli-tetrasomic line lacking the targeted chromosome, the recovered aberrations will be monosomic and hence easily detectable by PCR-based techniques. The selection of disomic/homozygous aberrations is performed following self-pollination of the plants carrying the aberrations, but the presence of the additional copy of a homoeologue inherited from the nulli-tetrasomic may complicate the transmission patterns of the targeted chromosome.

In this study, the 2C gametocidal system was used to develop a set of deletion lines for chromosome 3D in wheat to map the position of the *Ph2* gene. This gene was previously mapped using a *ph2a* mutant carrying a terminal deletion on chromosome 3D that was estimated to be approximately 80 Mb (Sutton et al., 2003).

## Materials and Methods

### Plant Material and Crosses

**Figure 1:**
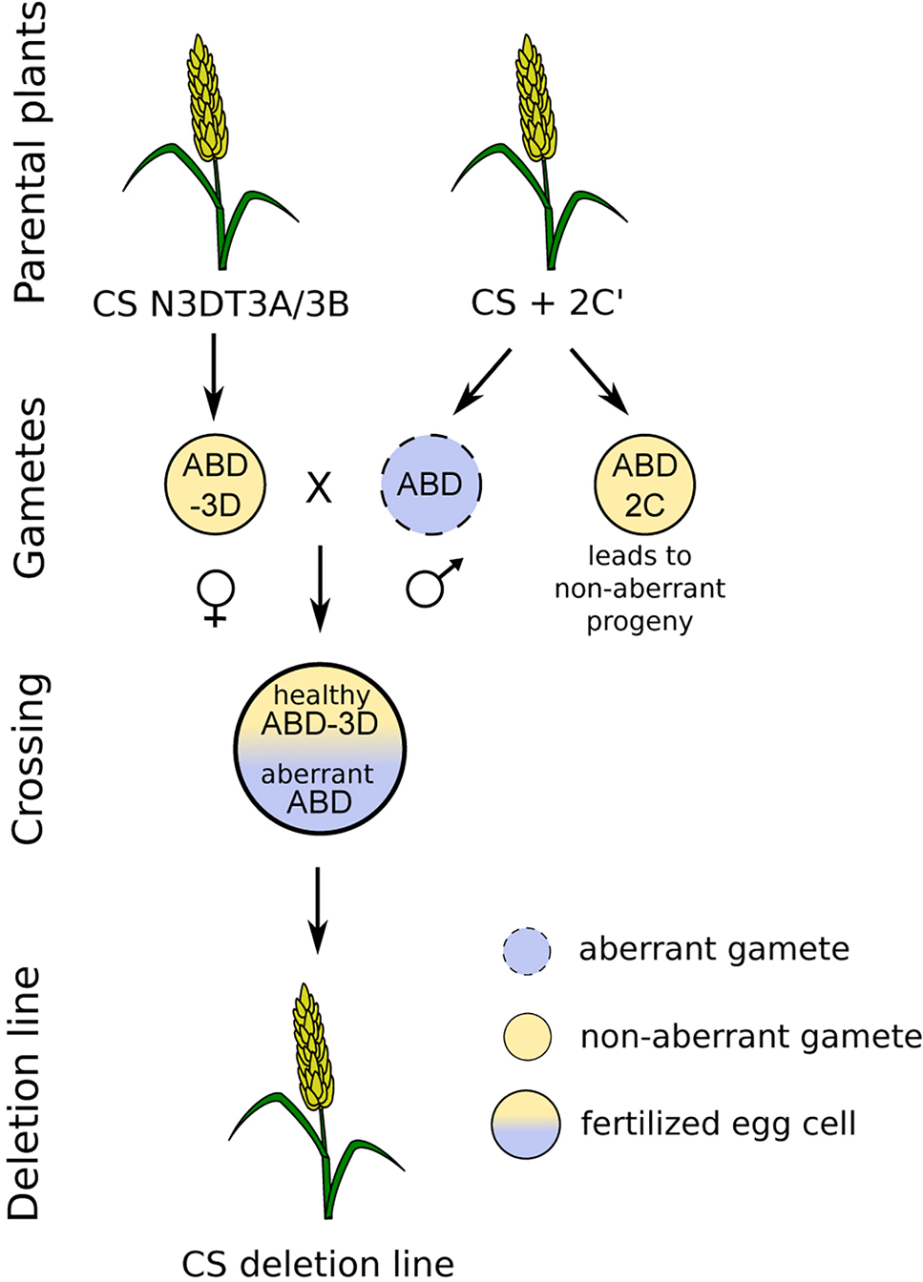
Crossing scheme used to develop deletion lines. The crossing was performed between nulli-tetrasomic plants lacking chromosome 3D with an extra chromosome pair 3A or 3B (female) and a monosomic addition line with an extra 2C chromosome from *Aegilops cylindrica* (male), with both on the ‘Chinese Spring' wheat background. The progeny contained a damaged set of chromosomes from the male parent and a healthy set lacking the 3D chromosome from the female parent.

The deletion lines were derived from crosses between the monosomic addition line of chromosome 2C from *A. cylindrica* in the hexaploid wheat cultivar Chinese Spring (CS) background (6x = 2n = 43; AABBDD + 2C′) used as male and the hexaploid CS wheat nulli-tetrasomic lines lacking chromosome 3D with tetrasomic constitution either for chromosome 3A or 3B (6x = 2n = 42; AABBDD − 3D″ + 3A″/3B″) used as female (**Figure 1**). The 2C gametocidal chromosome induces chromosomal breakages in gametes where it is not transferred, resulting mostly in terminal deletions. The crosses with nulli-tetrasomic lines lacking a pair of 3D chromosomes ensure that a potentially aberrant 3D chromosome from the 2C addition-line parent will be in the progeny in a monosomic state and that a deletion will not be masked by an entire 3D chromosome from the female parent. The plants were cultivated in growth chambers under the following conditions: a 16/8 h light/dark photoperiod, temperatures of 20 °C during the day and 16 °C at night, and 60% humidity.

### Identification of Plants With Deletion on the 3D Chromosome

The seeds acquired from the crosses were germinated in pots and cultivated for 2 weeks. Thereafter, DNA was isolated from a part of a young leaf by using a magnetic beads protocol (Sbeadx mini plant kit, LGC, Teddington, United Kingdom). The DNA was used to identify the deletion lines in the F1 generation. Molecular markers were designed for the distal ends of both arms of chromosome 3D, with a marker located in the centromeric area as a control for chromosome presence; primer details are shown in **Table 1**. The PCR was performed in 20 µl (1× PCR buffer, 1.5 mM MgCl, 200 µM dNTPs, 1 µM primers, 20 ng of DNA, 0.4 U/20 µl Taq DNA polymerase) under the following conditions: initial denaturation at 95 °C for 10 min; 35 cycles of denaturation at 95 °C for 30 s, annealing at 60 °C for 30 s, and elongation at 72 °C for 50 s; followed by a final extension at 72 °C for 10 min. The PCR was scored for the presence/absence of a specific product on 1.2% agarose gel. The plants carrying a deletion on chromosome 3D (lacking either or both 3DS and 3DL-specific PCR products) were replanted into larger pots and cultivated under the following conditions: a 16/8 h light/dark photoperiod, temperatures of 20 °C during the day and 16 °C at night, and 60% humidity. Plants were grown until seed harvest.

**Table 1:** Sequences and localization of primers used for identification of lines carrying a deletion on the short arm, long arm, or both arms of chromosome 3D.

Oligo ID	Sequence 5′–3′	Localization
3D_0.3Mb_F	TTAGTGGATCGAGGATTGTG	distal 3DS
3D_0.3Mb_R	TCGGTGACTAGTGTGTTTCTG	
3D_610.2Mb_F	GCAACAGAAGAAGAAAATACTGCT	distal 3DL
3D_610.2Mb_R	GTGCATCATATCTATGGTCTATC	
3D_253.4Mb_F	TATGCGTTTGGAGTAGTTCTTGT	3D centromere
3D_253.4Mb_R	CTCATCTCAGGCTGTCTAATTAA	

### Characterization of Sizes of Deletions

The deletion lines of chromosome 3D were characterized using a set of STS molecular markers covering the entire chromosome. In addition to the deletion lines, the X-ray-induced deletion mutant *ph2a* (Sears, 1950) was also characterized. Eighty-four was the final number of markers (**Supplementary Table 1**), of which 58 were on the short arm and 26 were on the long arm of the chromosome. The characterization was performed using presence/absence scoring and agarose gel electrophoresis separation as described above.

The primers for analysis were designed using the reference sequence of the wheat genome (IWGSC, 2018). The sequence was masked for annotated repetitive sequences. The loci for primer design were selected to cover the chromosome as evenly as possible with the priority in the distal 125 Mb of the short arm of chromosome 3D. The regions not masked with repeats (10–30 kb) were aligned using BLASTn against reference sequences of chromosomes 3A and 3B, and the corresponding regions were compared to depict the 3D-specific polymorphisms. Those polymorphisms were used to design 3D-specific primers (**Supplementary Table 1**). The primers were tested on *T. aestivum* ‘Chinese Spring’ as the positive control, a nulli-tetrasomic line lacking chromosome 3D as the negative control, and water as the blank.

### Identification of Deletion Lines With the 3D Chromosome in a Disomic State

Each deletion line was self-pollinated to increase seed stocks and to induce a disomic constitution of the 3D chromosome carrying a deletion. The upcoming generation comprised nullisomics, monosomics, and disomics for the analyzed chromosome. Therefore, screening with molecular markers was necessary to select the stable lines carrying the 3D chromosome with deletion in disomic constitution. First, the entire population was screened using the PCR marker on the centromere of the 3D chromosome (see above) to eliminate all nullisomics. The plants carrying the 3D chromosome were selected for droplet digital PCR (ddPCR) analysis. The ddPCR analysis was performed using ddPCR™ Supermix for Probes (no dUTP) (Bio-Rad, Hercules, USA) according to manufacturer's instructions with a 60 °C annealing/extension phase. The reference and target primers and TaqMan^®^ probes (Thermo Fisher Scientific, Waltham, USA) used for chromosomes 4A (disomic in all lines) and 3D are listed in the **Table 2**.

**Table 2:** Sequences of primers and probes used for determination of 3D chromosome number in the ddPCR assay. The TaqMan (taq) probes were either labelled by FAM (4A chromosome; used as a reference) or VIC (3D chromosome; target).

Oligo ID	Sequence and modifications 5'–3'	Amplicon length [bp]
Ta-4A_F	ATTTTGGGTCCTTGTTGTTATC	181
Ta-4A_R	ACACGCATGAAGTGTATAATGC
Ta-4A_taq	FAM-AAGAACTTCACACACGAACTCGGA-QSY
Ta-3D_F	CTCATCTCAGGCTGTCTAATTAA	167
Ta-3D_R	CATAGATCCCTCCTTGAAGGA
Ta-3D_taq	VIC-CCTCACTCAAGCACCACATCG-QSY

### Fluorescent *in Situ* Hybridization of Selected Lines

Selected lines carrying a deletion on the 3D chromosome were characterized cytogenetically using fluorescent *in situ* hybridization (FISH). Mitotic metaphase chromosomes were obtained from synchronized root tip meristems (Vrána et al., 2012). Synchronized roots were fixed in 90% ice-cold acetic acid for 10 min and then washed three times with 70% ethanol and stored at −20 °C in 70% ethanol. Chromosome preparations using the drop technique were performed according to Danilova et al. (2012). The individual chromosomes in the wheat karyotype were identified using the combination of two FISH probes: (GAA)_n_ microsatellite (FITC) and Afa repeat (Cy3) (Pedersen and Langridge, 1997; see **Supplementary Figure 1**). The probes were labeled *via* PCR, and FISH was performed under the conditions described in Kubaláková et al. (2003). The signals were observed using a Zeiss Axio Imager Z2 fluorescent microscope (Carl Zeiss, Jena, Germany) equipped with a CCD camera. At least five copies of the 3D chromosome per line were characterized by measurement of the deleted arm and whole chromosome length by using MicroImage software version 4.0 (Olympus, Shinjuku, Japan). The deletion size on chromosome 3D was estimated on the basis of the fragment length value (Endo and Gill, 1996).

## Results

From the F1 generation, 6169 seeds formed by crosses between the monosomic addition line of chromosome 2C from *A. cylindrica* and the nulli-tetrasomic lines (6x = 2n = 42; AABBDD − 3D″ + 3A″/3B″) were analyzed. The plants carrying a deletion on chromosome 3D were detected using STS markers designed for the terminal ends of 3D chromosomal arms. In total, 113 deletion lines were developed (**Supplementary Table 2**). All identified plants in the F1 generation carried a 3D chromosome with a deletion in monosomic constitution. More precisely, 43 (39.13%) of the lines carried a deletion on 3DS, 68 (60.87%) carried a deletion on 3DL, and two lines carried a deletion on both arms (for the schematic layout, see **Supplementary Figures 2**, **3**, and **4**). These numbers corresponded to the length–arm ratio of 0.393 (240 Mb) for the short arm and 0.607 (370 Mb) for the long arm (IWGSC, 2018).

A set of self-pollinations established a disomic constitution of deleted chromosomes in individual lines. The number of 3D copies was analyzed using a ddPCR protocol with specific primers and the TaqMan probe system comparing the number of events on the analyzed chromosome (3D) with that on the reference chromosome (4A). The disomic constitution of deletion chromosomes was successfully established in 102 of the 113 lines.

The whole set of deletion lines was characterized using 84 STS molecular markers evenly distributed along the entire 3D chromosome (**Supplementary Table 1**). The size of the deletions ranged from 6.5 to 357 Mb, and the size of the deletion bins (the region between two adjacent deletion breakpoints) ranged from 0.15 to 50 Mb. Some deletions seemed to have the same breakpoint; however, this was most likely caused by insufficient resolution of molecular markers in that particular region. The length of chromosome arm deletions and the number of missing genes in individual lines, as well as the differences in missing genes among the lines, are summarized in **Supplementary Table 2**.

The deletion lines of chromosome 3D were produced to map the position of the *Ph2* gene that was localized on this chromosome by Mello-Sampayo (1971). The position of this gene was further delimited using an X-ray-induced deletion mutant *ph2a* (Sears, 1982), and therefore, this mutant was included in the analysis as a control. The size of the deletion in the *ph2a* mutant was previously estimated to affect approximately 80 Mb in the terminal part of the 3DS using synteny with the rice chromosome (Sutton et al., 2003). However, the screening by molecular markers showed this deletion to be larger by approximately 40 Mb, because the breakage point was between 120 and 125 Mb.

**Figure 2:**
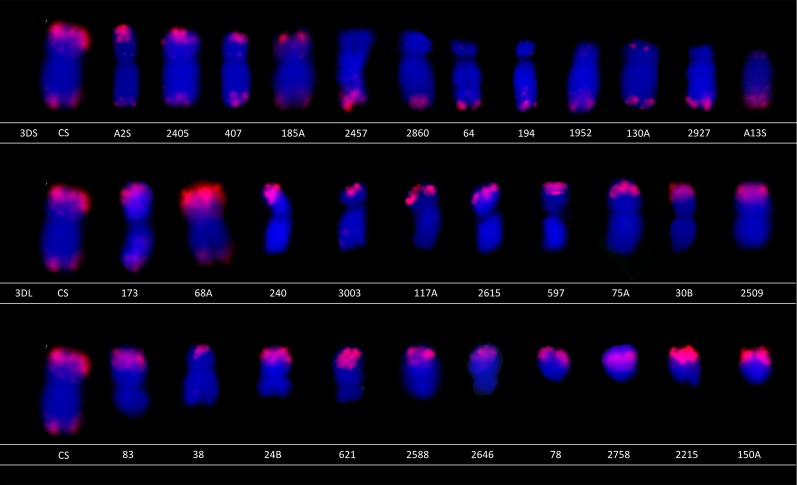
Characterization of selected lines using fluorescence *in situ* hybridization (FISH). The selected lines were characterized using FISH to confirm the deletion size. The chromosomes were labeled using (GAA)_n_ microsatellite (green) and Afa repeat (red) to distinguish chromosome 3D from other chromosomes. Note that only Afa repeat is present on chromosome 3D.

FISH analysis of selected deletion lines representing various lengths of deletions was performed to cytogenetically characterize the material (**Figure 2**). The 3D chromosome was identified using the Afa repeat family (Pedersen and Langridge, 1997). Among the 32 selected deletion lines, 12 had the breakage on the 3DS and 20 had the breakage on the 3DL. In all cases, the size of deletion determined by molecular markers was confirmed by cytogenetic observation.

## Discussion

The deletion lines were produced using the gametocidal system described by Endo and Gill (1996). The 2C gametocidal chromosome causes terminal chromosomal deletions in the gametes that lack it. However, these aberrations are usually not lethal because of the compensation by the other two homoeologous chromosomes. Thus, deletions can be transferred into progeny (Endo, 1988). Endo and Gill (1996) derived 436 plants *via* this system and characterized the deletions cytogenetically using a C-banding protocol. Of the 436 plants, 12 of them carried a deletion on chromosome 3D. In this study, 113 novel deletion lines for chromosome 3D were generated, increasing substantially the number of chromosome 3D deletion lines that are available for use in various applications.

Because the deletion lines were primarily produced to map the *Ph2* gene, the marker resolution was highest on the short arm of chromosome 3D. The 58 markers divided the short arm into segments ranging from 100 kb to a maximum of 29 Mb in the centromeric area. Owing to the high marker resolution, only a single or a few deletion breakpoints were assigned in each segment (**Supplementray Table 2**). The short arm of chromosome 3D comprises 1,949 annotated genes (IWGSC, 2018), and in this study, the estimated number of genes deleted in individual lines was 194–1,927, with the number of genes in each deletion bin ranging from 7 to 276. By contrast, the resolution achieved with 26 markers on the long arm of the chromosome was lower than that on the short arm, with segments ranging from 3 to 50 Mb. The long arm of chromosome 3D carries 3,369 genes (IWGSC, 2018), and the number of genes deleted in individual lines ranged from 306 to 3,351, with each deletion bin comprising between 76 and 468 genes (see **Supplementray Table 2**). The resolution of deletion bins in the area of the *Ph2* gene (distal 125 Mb of the short arm) ranged between 1.5 and 12 Mb, with an average of 6 Mb, limiting the number of potential candidate genes to between 7 and 276.

To produce single chromosome deletion lines *via* the 2C gametocidal system, a cross is performed with one parent a nulli-tetrasomic line lacking a chromosome of interest. The resulting progeny carry the deleted chromosome of interest in a monosomic constitution. Because the gametes produced by the progeny may or may not contain the deleted chromosome, the lines are unstable for direct use, making it unreliable material for seed stock enlargement, crossing, or physical gene mapping. Therefore, self-pollination of this material is recommended to accumulate the deleted chromosome in a disomic constitution. The self-pollination of a plant carrying a chromosome in the monosomic state can produce nullisomic, monosomic, or disomic progeny for the respective chromosome. However, the proportion of transmission to progeny of such a chromosome is shifted by various irregularities in univalent behavior in meiosis. In *Nicotiana tabacum*, the univalent elimination of different monosomic chromosomes occurs at the same frequency, fluctuating around 75% (Olmo, 1935). In wheat, however, the univalent elimination seems to have greater variability, depending on which chromosome is in a monosomic state (Morrison and Unrau, 1952; Tsunewaki and Heyne, 1960). In the material in this study, nullisomics occurred more frequently than expected in progeny of monosomic deletion lines. Univalent behavior during meiosis can explain the unexpected proportions of nullisomics, monosomics, and disomics in progeny. Because univalents lag behind the bivalents while being pulled to the poles at anaphase I, they are therefore excluded from newly formed nuclei and are preserved in the cytoplasm as micronuclei (Sears, 1950; Tsunewaki and Heyne, 1960).

In this study, the 2C gametocidal system was used to develop novel deletion lines for chromosome 3D in common wheat (Endo and Gill, 1996). The deleted chromosome was successfully fixed in disomic constitution in most of the material to ensure the stable inheritance of the chromosome of interest, which greatly improves further use of the deletion lines. The new material will be useful to clone genes of agronomic interest, such as *Ph2*, a gene involved in homoeologous pairing in bread wheat (Mello-Sampayo, 1971).

## Data Availability Statement

All datasets generated for this study are included in the article/**Supplementary Material**.

## Author Contributions

TE, PS, and JB designed the study. RS and TE crossed plants. RS, HS, and DV performed PCRs and ddPCR screening. MK and MM characterized the deletion lines using FISH. RS, PS, and JB wrote the manuscript. All authors approved the manuscript.

## Funding

This work was supported by the Czech Science Foundation (grant award 17-05341S) and the ERDF project “Plants as a tool for sustainable global development” (CZ.02.1.01/0.0/0.0/16_019/0000827). We acknowledge the Investment for the Future programme BREEDWHEAT (project ANR-10-BTBR-03) funded by the French Government and managed by the Research National Agency (ANR) for providing markers.

## Conflict of Interest

The authors declare that the research was conducted in the absence of any commercial or financial relationships that could be construed as a potential conflict of interest.
