# Supplementary material for: Development of Deletion Lines for Chromosome 3D of Bread Wheat
Source: Front Plant Sci. 2020 Jan 28;10:1756. doi: 10.3389/fpls.2019.01756 (PMC6997527; doi:10.3389/fpls.2019.01756)
Supplement: Supplementary file 1 [file DataSheet_1.pdf]

Supplementary data for

Development of deletion lines for chromosome 3D of bread wheat

Radim Svačina[1], Miroslava Karafiátová[1], Magdaléna Malurová[1], Heïdi Serra[2], Dominik Vítek[1], Takashi R. Endo[3], Pierre Sourdille[2], Jan Bartoš[1*]

[1]Institute of Experimental Botany, Czech Academy of Sciences, Centre of the Region Hana for Biotechnological and Agricultural Research, Olomouc, Czech Republic

[2]INRA, Génétique, Diversité, Ecophysiologie des Céréales, Clermont-Ferrand, France

[3]Faculty of Agriculture, Ryukoku University, Shiga, Japan

***Correspondence:**
Jan Bartoš
bartos@ueb.cas.cz

Supplementary figures

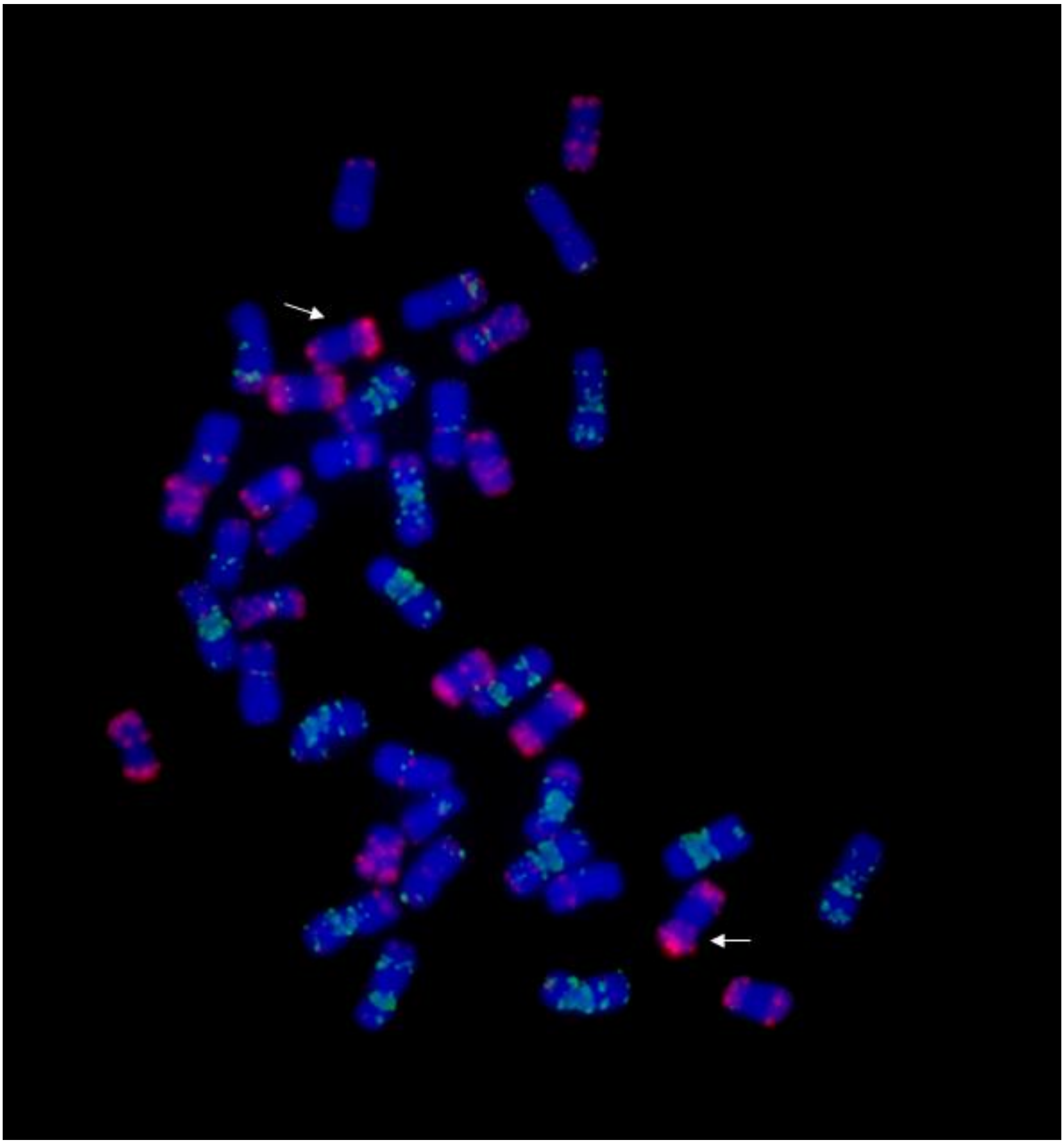

Supplementary Figure 1. Karyotype of bread wheat cv. Chinese Spring. The chromosomes were labelled using $(GAA)_n$ microsatellite (FITC; green) and Afa repeat (Cy3; red) to distinguish chromosome 3D from other chromosomes. The pair of 3D chromosomes is indicated using arrows.

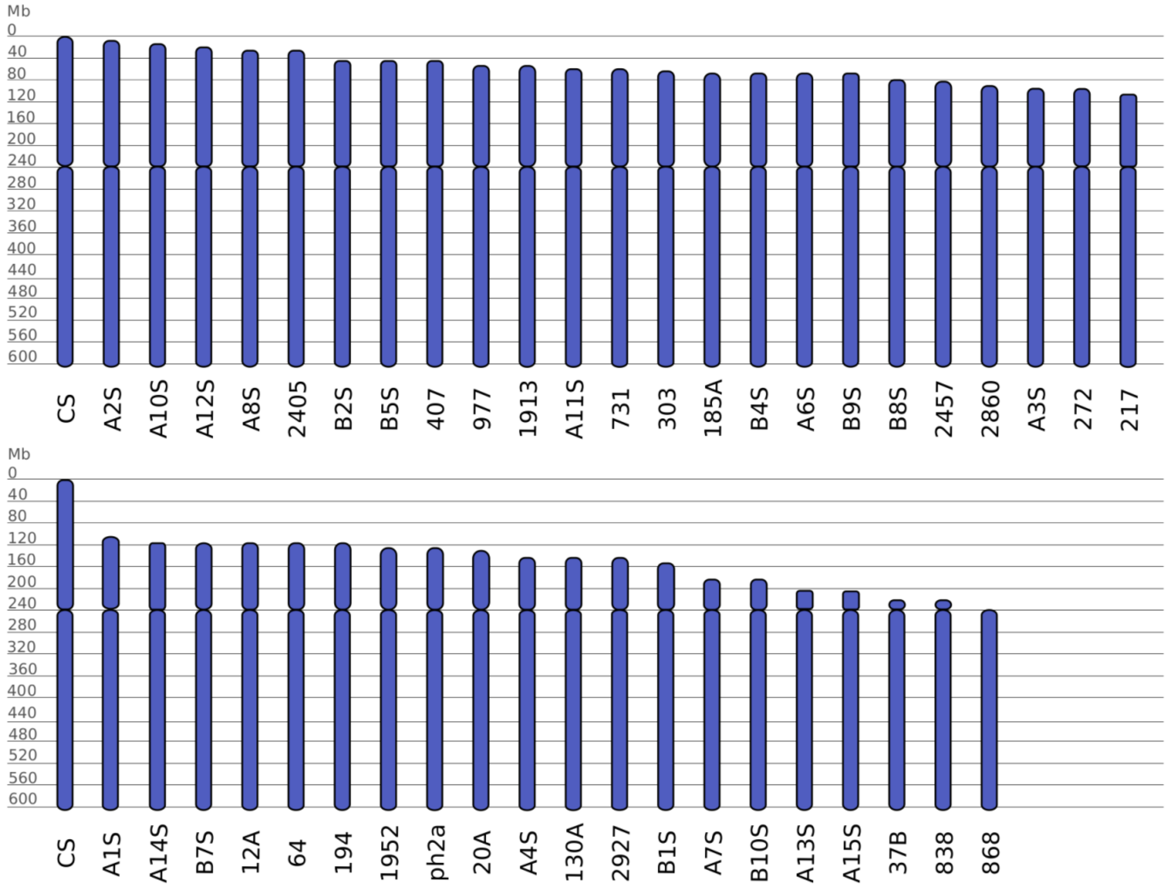

Supplementary Figure 2. The schematic layout of chromosome 3D in all lines carrying terminal deletions on a short arm. The y-axis indicates the size of chromosome (and deletion) in Mb.

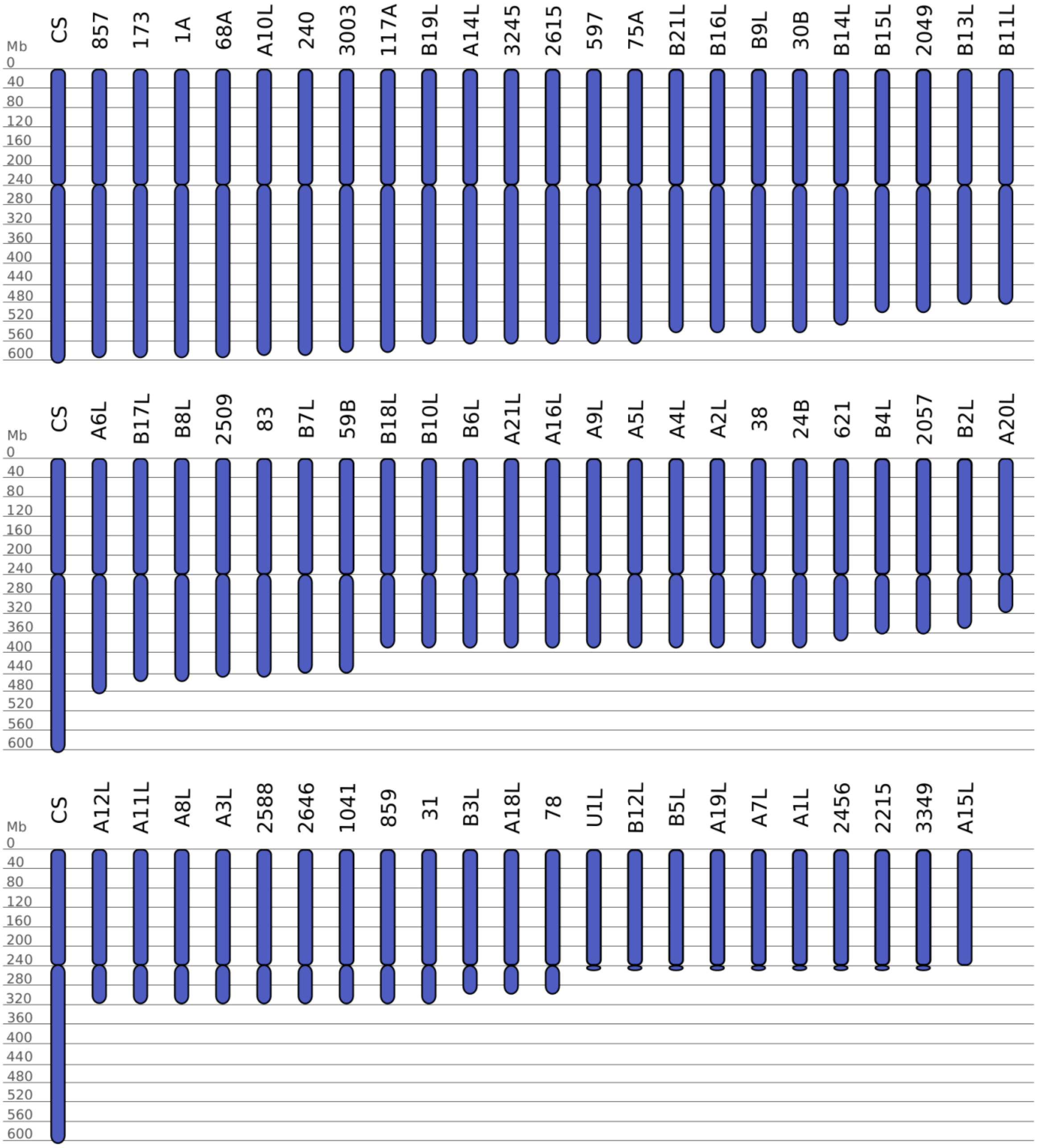

Supplementary Figure 3. The schematic layout of chromosome 3D in all lines carrying terminal deletions on a long arm. The y-axis indicates the size of chromosome (and deletion) in Mb.

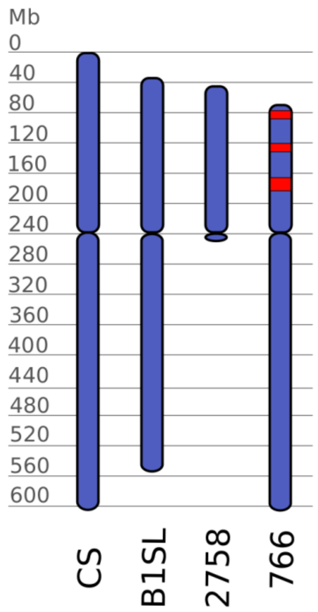

Supplementary Figure 4. The schematic layout of chromosome 3D in two lines carrying terminal deletions on both arms and an atypical line 766 carrying interstitial deletions. The y-axis indicates the size of chromosome (and deletion) in Mb.

Supplementary Tables

Supplementary Table 1. The sequences and positions of primers used for characterization of deletions on chromosome 3D. The positions correspond to pseudomolecule of chromosome 3D (IWGSC 2018)

Oligo ID	Chromosome arm	Sequence 5'-3'	Position [bp]	Amplicon size [bp]
3D_0.3Mb_F	3DS	TTAGTGGATCGAGGATTGTG	313,111 – 313,452	342
3D_0.3Mb_R	3DS	TCGGTGACTAGTGTGTTTCTG		
3D_3.2Mb_F	3DS	CACAAATCAGTTTACTATCATACCG	3,222,209 – 3,222,463	255
3D_3.2Mb_R	3DS	TAGAATTTATGTCGTAAATTTAATCTACC		
3D_5Mb_F	3DS	AGAGCTGCTGCTGGTGTTAC	5,005,285 – 5,005,746	420
3D_5Mb_R	3DS	ACCGTGCCTCTGACTGAAATTT		
3D_6.5Mb_F	3DS	TCGTCCACAAGAGCATCGAC	6,510,494 – 6,510,761	268
3D_6.5Mb_R	3DS	CCGAGCCGAACACATGGG		
3D_11.5Mb_F	3DS	CAGTCCTGTCCATGGTGTAG	11,461,616 – 11,461,969	314
3D_11.5Mb_R	3DS	GACTCTACAGGCGCCGATTT		
3D_13.3Mb_F	3DS	GGGAGTAAAGTCTATCAAGGCAA	13,297,388 – 13,297,736	349
3D_13.3Mb_R	3DS	GCAACATAAGTGGTCAATTATGCTA		
3D_19.3Mb_F	3DS	TCTAATGGGTCGATAAGAAGGTTT	19,308,909 – 19,309,287	333
3D_19.3Mb_R	3DS	GAGGAGAAGTACCTATGGTTGA		
3D_25.1Mb_F	3DS	GCACCTGAAGCTTAGATTTTG	25,119,750 – 25,119,994	245
3D_25.1Mb_R	3DS	GTATTACTCGAGGTGTGCCC		
3D_33.2Mb_F	3DS	AGATGAGGCCCATGGGAATC	33,164,040 – 33,164,392	311
3D_33.2Mb_R	3DS	ATTGGCCATGTAATGGACAGCA		
3D_38Mb_F	3DS	CAATGGATTTGGCACTTGTA	38,035,791 – 38,036,022	232
3D_38Mb_R	3DS	ACAAAGTACAGAAAAACACCATCT		
3D_43.8Mb_F	3DS	AGTGACAAGAGCCTCCTCGA	43,804,778 – 43,805,196	377
3D_43.8Mb_R	3DS	GCTTGCTTCTTGGTAAGGATAG		
3D_45.6Mb_F	3DS	AGGTTCGGTAACCATACGCC	45,624,543 – 45,624,988	446
3D_45.6Mb_R	3DS	CTACAATACTAAAACTATATCGCATG		
3D_49.8Mb_F	3DS	GGAGTTTAAGATCTTATTAAGAGATG	49,782,731 – 49,783,114	336
3D_49.8Mb_R	3DS	TTCAGACTTGCACTTGTACAGG		
3D_53.1Mb_F	3DS	ATGATAAACCTGACAAAGTTGGAC	53,104,805 – 53,105,002	151
3D_53.1Mb_R	3DS	CAAGTGAAACATCTCTTTCTGAC		
3D_58.6Mb_F	3DS	AGAGTTGAAATGGCTAATGCTACA	58,559,105 – 58,559,443	291
3D_58.6Mb_R	3DS	CATTTGCTTAAGACAACAGATTCC		
3D_62.9Mb_F	3DS	ATGGGACATAACCTATGTTGATAG	62,919,181 – 62,919,628	448
3D_62.9Mb_R	3DS	TACGTGAAATGTATAATCGTACGAAA		
3D_63.4Mb_F	3DS	TCTAGTCACTCATCGAGTCATG	63,356,418 – 63,357,041	624
3D_63.4Mb_R	3DS	GGGGATCTGCACAAAGTATAG		
3D_64.1Mb_F	3DS	ACTTCCAGTAAAATTTGGGG	64,081,522 – 64,081,707	186
3D_64.1Mb_R	3DS	TTATGTATGCATTGAATAGTCTCA		

**Oligo ID**	**Chromosome arm**	**Sequence 5'-3'**	**Position [bp]**	**Amplicon size [bp]**
3D_64.5Mb_F	3DS	CAGCCCAGTGAGATCAAA	64,481,718 – 64,481,851	134
3D_64.5Mb_R	3DS	AGAGAAAATGGCAACAGTCA		
3D_64.9Mb_F	3DS	TTTCTGTGAGAGCAATAAGAGG	64,858,404 – 64,858,655	252
3D_64.9Mb_R	3DS	CTTGTAGCTGGTAATGGACTATAGA		
3D_65.3Mb_F	3DS	CAAGAAAATCATAGGACCCTTG	65,315,330 – 65,315,593	264
3D_65.3Mb_R	3DS	GACCACCTATGAGATGATTCTG		
3D_65.6Mb_F	3DS	GTACCGTATTTGGAAACCTTC	65,616,212 – 65,616,591	380
3D_65.6Mb_R	3DS	GAGATCGATCGACACACAT		
3D_66.1Mb_F	3DS	CCCATCTCATTCCACAGATT	66,139,977 – 66,140,267	291
3D_66.1Mb_R	3DS	CTCCTCCATCTCCGATCT		
3D_66.4Mb_F	3DS	ATTTGGTATATCACTTCTGACAGG	66,405,526 – 66,405,776	251
3D_66.4Mb_R	3DS	TCTAACACACATAAAGTTAAGCTTCA		
3D_66.9Mb_F	3DS	GCGGTGCTATGCTTCCTA	66,890,697 – 66,890,790	94
3D_66.9Mb_R	3DS	GCCCGTCGACAGAGAAG		
3D_67.1Mb_F	3DS	GAGACGGTCCATTATCAAAATTCA	67,054,349 – 67,054,688	291
3D_67.1Mb_R	3DS	CCCAATCTATCATAATTTCCTCCAA		
3D_69.5Mb_F	3DS	TCAACTCATGTCATCCCC	69,487,272 – 69,487,553	282
3D_69.5Mb_R	3DS	AGCTGAGAGATAATGCACTGA		
3D_71.5Mb_F	3DS	TTGTAGTGCTTCACCGACTATA	71,513,709 – 71,513,857	149
3D_71.5Mb_R	3DS	AATCCTCCCTTACTACTAATGACAT		
3D_73.3Mb_F	3DS	CAAGCTAGAGCCAATAAGCTG	73,257,288 – 73,257,488	201
3D_73.3Mb_R	3DS	ATACATGTCCACAACATCAGC		
3D_74.1Mb_F	3DS	GAACTTCATAGGTGCCTTACA	74,054,542 – 74,054,920	379
3D_74.1Mb_R	3DS	AAATTACAGGGGAGCTAGAGAG		
3D_74.2Mb_F	3DS	GTTCGAAAGCAAGCAAAAC	74,164,513 – 74,164,781	269
3D_74.2Mb_R	3DS	AGTGAGTGACTTAAGCTAAACTAATTG		
3D_74.3Mb_F	3DS	TCCAGAGAAGGTCTTGCC	74,259,241 – 74,259,421	138
3D_74.3Mb_R	3DS	GCTTCTCTCAAGATAGCATAGTGTT		
3D_74.4Mb_F	3DS	TTTTCTGGTAGTATTGTGTCC	74,356,398 – 74,356,575	138
3D_74.4Mb_R	3DS	TATCCACACCGTCAACTGC		
3D_74.5Mb_F	3DS	CCCCTAAGCCTTTCTAAGTG	74,459,975 – 74,460,182	165
3D_74.5Mb_R	3DS	CAGTGCCTCAAGTATTTATTTGT		
3D_74.6Mb_F	3DS	GCAAATCTTGAGGAAATGAC	74,564,156 – 74,564,403	248
3D_74.6Mb_R	3DS	TATTCCTTCCCTATCTTGTTTTAAT		
3D_74.7Mb_F	3DS	ATCATCTCTGAGCAGATAACACT	74,664,473 – 74,664,717	245
3D_74.7Mb_R	3DS	CAATCGACCCTCATTAGTTC		
3D_75.6Mb_F	3DS	AAACAATTAAGACGTGTTCAGAAC	75,635,194 – 75,635,785	592
3D_75.6Mb_R	3DS	CATGAGGGAGAACCTTATGC		
3D_77.3Mb_F	3DS	CACAGATGAGTCCTTACTTATTTCT	77,341,428 – 77,341,676	249
3D_77.3Mb_R	3DS	CTGCTCCCAAACTAAGATTATG		
3D_78Mb_F	3DS	CATCTTCGATCTTCACCAAT	77,996,753 – 77,997,227	475
3D_78Mb_R	3DS	GATTGTGGTTCACTTCCAT		

Oligo ID	Chromosome arm	Sequence 5'-3'	Position [bp]	Amplicon size [bp]
3D_78.4Mb_F	3DS	CGTGCTACATGGCTTAGGA	78,365,563 – 78,365,817	255
3D_78.4Mb_R	3DS	CTCCGATCCATACTCCTGT		
3D_78.8Mb_F	3DS	ATGGAAGTTGTAATTACAACTATCG	78,762,387 – 78,762,606	220
3D_78.8Mb_R	3DS	ATACATTCAGCAATGTTAAGGAGT		
3D_79.2Mb_F	3DS	CCGGTACTAATGGTCTACCAG	79,248,033 – 79,248,475	397
3D_79.2Mb_R	3DS	GCCAGATATGACAGTTTATATCATG		
3D_82.1Mb_F	3DS	CATGTTAATAAGGATTGGTTCCCA	82,091,343 – 82,091,593	201
3D_82.1Mb_R	3DS	GAATACCTGATGTTATGTAAAGAGAA		
3D_85Mb_F	3DS	CGAAGATATACATTAGCCGACC	85,034,628 – 85,034,877	250
3D_85Mb_R	3DS	AAGACGGGTGATGAGGAGT		
3D_90.1Mb_F	3DS	TTCAGAGTGTGTTACTGACTCG	90,115,688 – 90,115,875	188
3D_90.1Mb_R	3DS	CGGATTGACTAGACCAATGA		
3D_95.5Mb_F	3DS	CAAATTTTGTACACATGTTCAACA	95,482,853 – 95,483,078	226
3D_95.5Mb_R	3DS	AGTATCAAACAATGTTGAATGGG		
3D_100.5Mb_F	3DS	GTCGTGTTGCCTTTTGAG	100,541,940 – 100,542,101	162
3D_100.5Mb_R	3DS	AGAAATGCAGAAGATATCAATGA		
3D_105.4Mb_F	3DS	CGGTTACGCCTAAGAGATACTC	105,420,612 – 105,420,913	302
3D_105.4Mb_R	3DS	GCCAGATACATGACCATAGAGAAT		
3D_110.4Mb_F	3DS	GATAGAGTTGATATGTACTCCTTC	110,409,273 – 110,409,622	304
3D_110.4Mb_R	3DS	GGATGAGCTATCATGACACAAG		
3D_116.3Mb_F	3DS	TGAGGTGTTTCCCTTTAGTTATC	116,330,986 – 116,331,337	352
3D_116.3Mb_R	3DS	CAATAAATGTTGCAGTTCGTAG		
3D_120.1Mb_F	3DS	CACTATACCTTAAGCTTTCTCAGC	120,118,935 – 120,119,064	130
3D_120.1Mb_R	3DS	ATTACTAGCTGGATGTTGTCGT		
3D_125.3Mb_F	3DS	CCGTGTGTTGTGGGATACT	125,283,114 – 125,283,517	404
3D_125.3Mb_R	3DS	GCCATTTTTCCTTGACCTT		
3D_130.5Mb_F	3DS	TCTGATCTAATGAATGTCTCCAAC	130,498,053 – 130,498,474	422
3D_130.5Mb_R	3DS	TGAACTAAAATTTGAGCGTTATATCG		
3D_142.6Mb_F	3DS	CGAACCTGGAGGATCCAGT	142,617,171 – 142,617,500	330
3D_142.6Mb_R	3DS	CAATGTTTTACATTCCGCTGCTA		
3D_152.8Mb_F	3DS	GCAACTGAGGCATATCACCG	152,846,731 – 152,846,938	208
3D_152.8Mb_R	3DS	CCCATGGAAATGTGAAACTATCAA		
3D_181.6Mb_F	3DS	CACAAAAGAATAAAGATGTCCAAAGA	181,564,167 – 181,564,488	322
3D_181.6Mb_R	3DS	TCATGTCTATGACCCTAGGGAA		
3D_200.4Mb_F	3DS	GCAATTCGTGCTTTATTTAGGTA	200,378,253 – 200,378,539	287
3D_200.4Mb_R	3DS	GAGCAAATGGAAGAGAAGAAAAT		
3D_220.6Mb_F	3DS	GCATTGTCGTTAGCAATACAAT	220,640,507 – 220,640,735	229
3D_220.6Mb_R	3DS	CACATATTTTCCAGACAGGTG		
3D_253.4Mb_F	3DL	TATGCGTTTGGAGTAGTTCTTGT	253,391,812 – 253,392,248	391
3D_253.4Mb_R	3DL	CTCATCTCAGGCTGTCTAATTAA		
3D_260.3Mb_F	3DL	AAGAAGGCGATGAGCAGCCAA	260,344,666 – 260,345,083	418
3D_260.3Mb_R	3DL	GCTGATGGTGGCCGACTTC		

Oligo ID	Chromosome arm	Sequence 5'-3'	Position [bp]	Amplicon size [bp]
3D_291.9Mb_F	3DL	ACCGCTCATTGATTAATGAGTCTA	291,922,309 – 291,922,542	187
3D_291.9Mb_R	3DL	GAACTTTCTTCTAGGTTGCCATG		
3D_297.9Mb_F	3DL	GATGGGAGGAACAACTGGATC	297,919,462 – 297,919,713	252
3D_297.9Mb_R	3DL	CTATTTTCATCGACAACATGGCTTT		
3D_300.9Mb_F	3DL	CGAACTGTCCAGAAGTGACC	300,870,277 – 300,870,636	320
3D_300.9Mb_R	3DL	GGAAGAACCCAAATGCACCG		
3D_321.6Mb_F	3DL	GCCAAATTAACTCGGCCTTGTAT	321,580,894 – 321,581,000	107
3D_321.6Mb_R	3DL	GCTCGCTGTGCACCCTTG		
3D_355.4Mb_F	3DL	TAGGCCACGCTTTCCCCTA	355,372,772 – 355,372,952	181
3D_355.4Mb_R	3DL	GCGAGAACGAGAATGAGCAAAT		
3D_365.3Mb_F	3DL	CGCTCCTAAGCCCAAGAAACT	365,293,244 – 365,293,510	227
3D_365.3Mb_R	3DL	GGGCTTAGTGGGGCCGTAA		
3D_379.4Mb_F	3DL	GTCATTTGTACAAGCCCGTAC	379,415,556 – 379,415,765	170
3D_379.4Mb_R	3DL	ACCGAACGCTGCTACAAGG		
3D_394.9Mb_F	3DL	TTTGAGCATATATATAGCCCACAAAT	394,938,269 – 394,938,625	357
3D_394.9Mb_R	3DL	GATTTCATATGAAACTGTGGTAGTA		
3D_445.3Mb_F	3DL	GTGTAAATGATTATCATTTGGTTCTTAC	445,308,079 – 445,308,504	379
3D_445.3Mb_R	3DL	CTTTTCCGGACGGAGGGAG		
3D_455.1Mb_F	3DL	GACGCTATGGGCAACACGTT	455,115,226 – 455,115,401	176
3D_455.1Mb_R	3DL	TCTAAGTGAGGCCAGTACTGAT		
3D_463.1Mb_F	3DL	GCCAACATTGAGAGTGGCCT	463,093,990 – 463,094,160	171
3D_463.1Mb_R	3DL	TCATCGGCATGCTCTCTGCT		
3D_476.5Mb_F	3DL	GCGCTTGCTGGTATTTCCATTT	476,513,555 – 476,513,743	142
3D_476.5Mb_R	3DL	CTATAGTATACTCCTAACAAGCTAG		
3D_488.2Mb_F	3DL	GAAGGAGCTCTCATGATCTTTC	488,246,272 – 488,246,492	177
3D_488.2Mb_R	3DL	CAAGAGAGTTCTGAAGACAACC		
3D_504Mb_F	3DL	TGCTCCTGGATCTAACTTGCA	504,045,234 – 504,045,527	294
3D_504Mb_R	3DL	TTCCGAACAGTTTGCCTTCTC		
3D_510.8Mb_F	3DL	CATTCTGATGAATATTTATTACGAATACT	510,836,057 – 510,836,426	321
3D_510.8Mb_R	3DL	CCCTTGACGAAAGCACCTCT		
3D_529.8Mb_F	3DL	GGACACACACATAATATTTGCAGT	529,783,437 – 529,783,643	207
3D_529.8Mb_R	3DL	CGAATTTGCTGTAAACAAGCATGA		
3D_538.4Mb_F	3DL	AAAGCTCGGAAAGGGATGGG	538,406,637 – 538,406,954	271
3D_538.4Mb_R	3DL	ATTAATCCAAGCATATAAAACCCATGA		
3D_545.9Mb_F	3DL	TGACATCAACAATTGGCACCAAAT	545,928,824 – 545,929,156	333
3D_545.9Mb_R	3DL	CTAATTACTACTGAATCAACGTCCA		
3D_556.8Mb_F	3DL	CTTTGACTTGTTATACTAAGCCGA	556,802,010 – 556,802,214	205
3D_556.8Mb_R	3DL	GTCCGATCATAACTTCCGTCC		
3D_569.9Mb_F	3DL	GTAACTCCAAGGGGTGTACAG	569,879,809 – 569,880,027	173
3D_569.9Mb_R	3DL	CTGCTGTAGTGTTGCTTTGTATATT		
3D_587Mb_F	3DL	TCATTCTCTACCATGCCTGGAA	586,952,601 – 586,953,012	364
3D_587Mb_R	3DL	AATATCAAGATGGCCAAGTTTATGAA		

**Oligo ID**	**Chromosome arm**	**Sequence 5'-3'**	**Position [bp]**	**Amplicon size [bp]**
3D_594Mb_F	3DL	ATCTGGTATATATTGAGTTGAGCC	593,971,624 – 593,971,982	359
3D_594Mb_R	3DL	AAAAACTATATGCCATCACCATGC		
3D_598.5Mb_F	3DL	GTAATATGTAAAAACTGAACTTGTTCG	598,540,714 – 598,540,926	213
3D_598.5Mb_R	3DL	AATCTACCGTGATAGCTTCCGT		
3D_610.2Mb_F	3DL	GCAACAGAAGAAGAAAATACTGCT	610,194,433 – 610,194,748	269
3D_610.2Mb_R	3DL	GTGCATCATATCTATGGTCTATC		

Supplementary Table 2. Summary information about all derived deletion lines. Maximal length of deletion is estimated according to the first positive marker. The table also shows a number of missing genes in individual lines and a difference in gene number between adjoining lines.

ID	Affected arm	Max breakage position [bp]	First positive marker ID	Monosomic /disomic 3D	No. of missing genes on 3D	No. of genes differing from adjoining line
A2S	3DS	6,510,494	3D_6.5Mb	Disomic	194	172
A10S	3DS	13,297,388	3D_13.3Mb	Disomic	366	120
A12S	3DS	19,308,909	3D_19.3Mb	Disomic	486	77
A8S	3DS	25,119,750	3D_25.1Mb	Disomic	563	276
2405	3DS	25,119,750	3D_25.1Mb	Disomic	563	276
B2S	3DS	43,804,778	3D_43.8Mb	Disomic	839	134
B5S	3DS	43,804,778	3D_43.8Mb	Disomic	839	134
407	3DS	43,804,778	3D_43.8Mb	Disomic	839	134
977	3DS	53,104,805	3D_53.1Mb	Disomic	973	58
1913	3DS	53,104,805	3D_53.1Mb	Disomic	973	58
A11S	3DS	58,559,105	3D_58.6Mb	Disomic	1031	26
731	3DS	58,559,105	3D_58.6Mb	Disomic	1031	26
303	3DS	62,919,181	3D_62.9Mb	Disomic	1057	7
185A	3DS	64,081,522	3D_64.1Mb	Disomic	1064	17
B4S	3DS	64,081,522	3D_64.1Mb	Disomic	1064	17
A6S	3DS	65,616,212	3D_65.6Mb	Disomic	1081	10
B9S	3DS	67,054,349	3D_67.1Mb	Disomic	1091	85
B8S	3DS	79,248,033	3D_79.2Mb	Disomic	1176	28
2457	3DS	82,091,343	3D_82.1Mb	Disomic	1204	55
2860	3DS	90,115,688	3D_90.1Mb	Disomic	1259	67
A3S	3DS	95,482,853	3D_95.5Mb	Disomic	1326	59
272	3DS	95,482,853	3D_95.5Mb	Disomic	1326	59
217	3DS	105,420,612	3D_105.4Mb	Disomic	1385	86
A1S	3DS	105,420,612	3D_105.4Mb	Disomic	1385	86
A14S	3DS	116,330,986	3D_116.3Mb	Disomic	1471	48
B7S	3DS	116,330,986	3D_116.3Mb	Disomic	1471	48
12A	3DS	116,330,986	3D_116.3Mb	Disomic	1471	48
64	3DS	116,330,986	3D_116.3Mb	Monosomic	1471	48
194	3DS	116,330,986	3D_116.3Mb	Disomic	1471	48
1952	3DS	125,283,114	3D_125.3Mb	Disomic	1519	25
ph2a	3DS	125,283,114	3D_125.3Mb	-	1519	25
20A	3DS	130,498,053	3D_130.5Mb	Disomic	1544	83
A4S	3DS	142,617,171	3D_142.6Mb	Disomic	1627	36
130A	3DS	142,617,171	3D_142.6Mb	Disomic	1627	36
2927	3DS	142,617,171	3D_142.6Mb	Disomic	1627	36
B1S	3DS	152,846,731	3D_152.8Mb	Disomic	1663	191
A7S	3DS	181,564,167	3D_181.6Mb	Disomic	1854	49
B10S	3DS	181,564,167	3D_181.6Mb	Monosomic	1854	49
A13S	3DS	200,378,253	3D_200.4Mb	Monosomic	1903	24
A15S	3DS	200,378,253	3D_200.4Mb	Monosomic	1903	24
37B	3DS	220,640,507	3D_220.6Mb	Disomic	1927	-

ID	Affected arm	Max breakage position [bp]	First positive marker ID	Monosomic /disomic 3D	No. of missing genes on 3D	No. of genes differing from adjoining line
838	3DS	220,640,507	3D_220.6Mb	Disomic	1927	-
868	3DS	centromere	-	Disomic	-	-
A15L	3DL	centromere	-	Monosomic	-	-
3349	3DL	253,392,248	3D_253.4Mb	Disomic	3351	-
2215	3DL	253,392,248	3D_253.4Mb	Disomic	3351	-
2456	3DL	253,392,248	3D_253.4Mb	Disomic	3351	-
A1L	3DL	253,392,248	3D_253.4Mb	Disomic	3351	-
A7L	3DL	253,392,248	3D_253.4Mb	Disomic	3351	-
A19L	3DL	253,392,248	3D_253.4Mb	Disomic	3351	-
B5L	3DL	253,392,248	3D_253.4Mb	Disomic	3351	-
B12L	3DL	253,392,248	3D_253.4Mb	Disomic	3351	-
U1L	3DL	253,392,248	3D_253.4Mb	Disomic	3351	-
78	3DL	300,870,636	3D_300.9Mb	Disomic	3184	167
A18L	3DL	300,870,636	3D_300.9Mb	Disomic	3184	167
B3L	3DL	300,870,636	3D_300.9Mb	Disomic	3184	167
31	3DL	321,581,000	3D_321.6Mb	Disomic	3061	123
859	3DL	321,581,000	3D_321.6Mb	Disomic	3061	123
1041	3DL	321,581,000	3D_321.6Mb	Disomic	3061	123
2646	3DL	321,581,000	3D_321.6Mb	Disomic	3061	123
2588	3DL	321,581,000	3D_321.6Mb	Disomic	3061	123
A3L	3DL	321,581,000	3D_321.6Mb	Disomic	3061	123
A8L	3DL	321,581,000	3D_321.6Mb	Disomic	3061	123
A11L	3DL	321,581,000	3D_321.6Mb	Disomic	3061	123
A12L	3DL	321,581,000	3D_321.6Mb	Monosomic	3061	123
A20L	3DL	321,581,000	3D_321.6Mb	Disomic	3061	123
B2L	3DL	355,372,952	3D_355.4Mb	Disomic	2858	203
2057	3DL	365,293,510	3D_365.3Mb	Disomic	2771	87
B4L	3DL	365,293,510	3D_365.3Mb	Disomic	2771	87
621	3DL	379,415,765	3D_379.4Mb	Disomic	2671	100
24B	3DL	394,938,625	3D_394.9Mb	Disomic	2551	120
38	3DL	394,938,625	3D_394.9Mb	Disomic	2551	120
A2L	3DL	394,938,625	3D_394.9Mb	Disomic	2551	120
A4L	3DL	394,938,625	3D_394.9Mb	Disomic	2551	120
A5L	3DL	394,938,625	3D_394.9Mb	Disomic	2551	120
A9L	3DL	394,938,625	3D_394.9Mb	Disomic	2551	120
A16L	3DL	394,938,625	3D_394.9Mb	Disomic	2551	120
A21L	3DL	394,938,625	3D_394.9Mb	Monosomic	2551	120
B6L	3DL	394,938,625	3D_394.9Mb	Disomic	2551	120
B10L	3DL	394,938,625	3D_394.9Mb	Disomic	2551	120
B18L	3DL	394,938,625	3D_394.9Mb	Disomic	2551	120
59B	3DL	445,308,504	3D_445.3Mb	Disomic	2083	468
B7L	3DL	445,308,504	3D_445.3Mb	Disomic	2083	468
83	3DL	455,115,401	3D_455.1Mb	Monosomic	1992	91
2509	3DL	455,115,401	3D_455.1Mb	Disomic	1992	91

ID	Affected arm	Max breakage position [bp]	First positive marker ID	Monosomic /disomic 3D	No. of missing genes on 3D	No. of genes differing from adjoining line
B8L	3DL	463,094,160	3D_463.1Mb	Disomic	1900	92
B17L	3DL	463,094,160	3D_463.1Mb	Disomic	1900	92
A6L	3DL	488,246,492	3D_488.2Mb	Monosomic	1677	223
B11L	3DL	488,246,492	3D_488.2Mb	Disomic	1677	223
B13L	3DL	488,246,492	3D_488.2Mb	Disomic	1677	223
2049	3DL	504,045,527	3D_504Mb	Disomic	1542	135
B15L	3DL	504,045,527	3D_504Mb	Monosomic	1542	135
B14L	3DL	529,783,643	3D_529.8Mb	Disomic	1254	288
30B	3DL	545,929,156	3D_545.9Mb	Disomic	1118	136
B9L	3DL	545,929,156	3D_545.9Mb	Disomic	1118	136
B16L	3DL	545,929,156	3D_545.9Mb	Disomic	1118	136
B21L	3DL	545,929,156	3D_545.9Mb	Disomic	1118	136
75A	3DL	569,880,027	3D_569.9Mb	Disomic	789	329
597	3DL	569,880,027	3D_569.9Mb	Disomic	789	329
2615	3DL	569,880,027	3D_569.9Mb	Disomic	789	329
3245	3DL	569,880,027	3D_569.9Mb	Disomic	789	329
A14L	3DL	569,880,027	3D_569.9Mb	Monosomic	789	329
B19L	3DL	569,880,027	3D_569.9Mb	Disomic	789	329
117A	3DL	586,953,012	3D_587Mb	Disomic	515	274
3003	3DL	586,953,012	3D_587Mb	Disomic	515	274
240	3DL	593,971,982	3D_594Mb	Disomic	382	133
A10L	3DL	593,971,982	3D_594Mb	Disomic	382	133
68A	3DL	598,540,926	3D_598.5Mb	Disomic	306	76
1A	3DL	598,540,926	3D_598.5Mb	Disomic	306	76
173	3DL	598,540,926	3D_598.5Mb	Disomic	306	76
857	3DL	598,540,926	3D_598.5Mb	Disomic	306	76
B1SL	3DS	33,164,040	3D_33.2Mb	Disomic	1665	-
	3DL	556,802,214	3D_556.8Mb			
2758	3DS	43,804,778	3D_43.8Mb	Disomic	4190	-
	3DL	253,392,248	3D_253.4Mb			
766	3DS	interstitial,deletions	3D_69.5Mb; 3D_77.3Mb; 3D_85.0Mb; 3D_120.1Mb; 3D_130.5Mb	Disomic	-	
